# Photothermal Therapy-Induced Immunogenic Cell Death Synergistically Enhances the Therapeutic Effect of Immune Checkpoint Inhibitors

**DOI:** 10.3390/cancers18020287

**Published:** 2026-01-16

**Authors:** Shogo Yasuda, Yui Horikawa, Mei Ohashi, Mai Amou, Taisei Kanamori, Duan Runjing, Yuta Tamemoto, Wei Xu, Takuro Niidome, Akihiro Hisaka, Hiroto Hatakeyama

**Affiliations:** 1 Laboratory of DDS Design and Drug Disposition, Graduate School of Pharmaceutical Sciences, Chiba University, 1-8-1 Inohana, Chuo-ku, Chiba-City 260-8675, Chiba, Japan; yasudashogo424@gmail.com (S.Y.);; 2 Laboratory of Clinical Pharmacology and Pharmacometrics, Graduate School of Pharmaceutical Sciences, Chiba University, 1-8-1 Inohana, Chuo-ku, Chiba-City 260-8675, Chiba, Japan; 3 Faculty of Advanced Science and Technology, Kumamoto University, 2-39-1 Kurokami, Chuo-ku, Kumamoto-City 860-8555, Kumamoto, Japan; xuwei@kumamoto-u.ac.jp (W.X.); niidome@kumamoto-u.ac.jp (T.N.); 4 Center of Quantum Life Science for Structural Therapeutics (cQUEST), Chiba University, 1-33 Yayoi-cho, Inage-ku, Chiba-City 263-8522, Chiba, Japan

**Keywords:** photothermal therapy, immunogenic cell death, damage-associated molecular patterns, immune checkpoint inhibitor

## Abstract

**Background/Objectives:** To improve the response rate of immune checkpoint inhibitors (ICIs), inducing immunogenic cell death (ICD) is a promising approach. Photothermal therapy (PTT) induces immunogenic cell death and activates anti-tumor immunity. While there are various ICD inducers, the difference in ICD induction by various modalities is poorly understood. In this study, we found previously unrecognized advantages of PTT compared to anti-cancer drugs and showed the usefulness of PTT as an anti-cancer drug-free approach to be combined with immunotherapy. **Methods:** Gold nanorods were synthesized as photothermal agents and added to culture medium or locally administered to tumor tissues. Mitoxantrone (MIT), an ICD inducer, and cisplatin (CDDP), a non-ICD inducer, were compared with PTT. To assess the induction of ICD, the subcellular localization and amounts of high mobility group box 1 (HMGB1) and calreticulin (CRT) were observed using immunofluorescent staining. FM3A tumor-bearing mice were treated with PTT or anti-cancer drugs, and cell death and DAMPs localization in tumor tissues were analyzed. Also, the supra-additive effect of PTT on ICI was observed. Tumor-infiltrating CD8^+^ T cells were examined to evaluate the immune status in tumor tissues. **Results:** In vivo assays showed that PTT induces HMGB1 release and increased expression of CRT on the cell membrane. Moreover, PTT showed a supra-additive effect in terms of therapeutic effect and anti-tumor activation when combined with an immune checkpoint inhibitor. **Conclusions:** In this study, we demonstrated that PTT induced ICD-related signaling and improved the response rate of ICI, which means PTT is a promising combination therapy with ICI.

## Simple Summary

Photothermal therapy (PTT), which kills cancer cells through thermal stress, can induce immunogenic cell death (ICD), thereby enhancing the therapeutic effect of immunotherapy. Although several ICD inducers exist, their relative ICD induction capabilities remain unclear. The purpose of this study is comparing the ICD-induction ability of anti-cancer drugs and PTT and explore the therapeutic potential of PTT combined with ICIs. In vitro assays demonstrated that cisplatin, a non-ICD inducer, failed to trigger high mobility group box protein 1 (HMGB1) release or upregulation of calreticulin (CRT) expression; whereas, mitoxantrone, an ICD inducer, promoted HMGB1 release but not CRT expression. In contrast, PTT induced both HMGB1 release and increased CRT expression. Furthermore, PTT promoted infiltration of CD8^+^ T cells in tumor tissues and exhibited a synergistic effect when combined with ICIs. These results suggest that PTT could be a drug-free, minimally invasive approach for ICD induction that can be combined with immunotherapy.

## 1. Introduction

Immune checkpoint inhibitors (ICIs)—such as monoclonal antibodies targeting cytotoxic T-lymphocyte-associated protein 4 (CTLA-4), programmed cell death protein-1 (PD-1), and its ligand PD-L1—have been approved for treating diverse cancers [1]. ICIs have revolutionized cancer therapy owing to their novel characteristic of regulating antitumor immunity and producing remarkable antitumor effects in a proportion of patients with advanced or refractory cancers. However, the objective response rate (ORR) of ICIs varies substantially across cancer types. For example, reported ORRs are approximately 20–25% in renal cell carcinoma and 5% in breast cancer, and a large proportion of patients with cancer are unresponsive to ICI treatment [2]. Although several biomarkers have been identified to distinguish responders from non-responders, ICIs exhibit enhanced therapeutic efficacy in patients with DNA mismatch repair-deficient or microsatellite instability-high (dMMR/MSI-H) status in colorectal cancer and other cancers [2,3,4]—the ORR still remains below 50%. Therefore, combination therapies are required to improve therapeutic outcomes.

Numerous clinical trials have explored combination therapies to enhance sensitivity to ICIs, aimed at modulating the tumor microenvironment (TME), particularly by inducing immunogenic cell death (ICD), which activates systemic antitumor immunity [5]. Various therapeutic modalities, including chemotherapy, radiotherapy, and photodynamic therapy (PDT), induce ICD [6]. In addition to these, photothermal therapy (PTT) has recently gained attention as an ICD-inducing modality. PTT mainly relies on optical energy-absorbing agents called photothermal agents, capable of converting optical energy—such as radio waves, microwaves [7,8,9], and near-infrared (NIR) radiation [10]—into heat. PTT is a minimally invasive local treatment modality with low toxicity. Moreover, PTT-induced local hyperthermia can induce ICD and trigger systemic adaptive immune responses [11,12]. Therefore, PTT-induced ICD may promote the cancer-immunity cycle, potentially improving the response rate of ICIs when used in combination.

ICD is characterized by the release of damage-associated molecular patterns (DAMPs), such as high-mobility group box 1 (HMGB1), calreticulin (CRT), heat shock proteins (HSPs), and ATP [6,13]. The induction of ICD is closely linked to cytotoxic mechanisms and cell death morphology. Although PTT is a promising approach to induce ICD, it remains unclear whether the DAMPs released by PTT are as effective as those generated by other conventional modalities such as chemotherapy. Understanding the differences in the characteristics of ICD induced by different modalities may unveil previously unrecognized advantages of PTT-induced ICD.

Therefore, a comprehensive understanding of the ICD profiles induced by different modalities is required. In this study, we investigated the release of DAMPs induced by multiple modalities and demonstrated that PTT elicits a more efficient ICD response than conventional anti-cancer drugs. Our findings highlight the unique profile of PTT-induced ICD-associated signaling and its potential usefulness as an anti-cancer drug-free approach in combination with immunotherapy.

## 2. Materials and Methods

### 2.1. Preparation of Gold Nanorods

Gold nanorods were purchased from Dai Nippon Toryo Co., Ltd. (Osaka, Japan). The preparation of gold nanorods was conducted according to previous reports [14]. Excess cetyltrimethylammonium bromide (CTAB) used as a stabilizer in the suspension was removed by centrifugation of the gold nanorods at 12,000× *g* for 10 min and resuspending them in water (two cycles). The gold nanorods were then modified with thiol-terminated polyethylene glycol (SH-PEG, MW 5000 Da) to improve their biocompatibility and stability. SH-PEG was added at a PEG/gold molar ratio of 1.5, and the suspension was stirred overnight at room temperature (25 °C). To remove unreacted SH-PEG, a dialysis membrane (MWCO 12,000 Da) was used, and the dialysis water was changed three times daily for three days.

### 2.2. Cell Culture

The murine mammary carcinoma cell line, FM3A, was obtained from the RIKEN Cell Bank (Tsukuba, Japan). Murine colon adenocarcinoma MC38 was purchased from Kerafast (Boston, MA, USA). FM3A cells were cultured in RPMI 1640 (Nacalai Tesque, Kyoto, Japan, #06261-65) supplemented with 10% fetal bovine serum (FBS; Biowest, Nuaille, France, #S1820) and 1% penicillin and streptomycin (Nacalai Tesque, #26253-84) in 5% CO_2_ at 37 °C. MC38 cells were cultured in DMEM with 10% fetal bovine serum (FBS), 10 mM Hepes, 1% non-essential amino acids, 1 mM sodium pyruvate L-glutamine, and 1% penicillin and streptomycin.

### 2.3. In Vitro PTT Treatment

Cells were seeded in a 24-well plate (3.0–10.0 × 10^4^ cells/mL/well) at an adequate distance to remove the effect of spillover and incubated with gold nanorods (20 µg Au/well). After that, the medium was irradiated with NIR light using a MAX-303 (Asahi Spectra, Tokyo, Japan) equipped with a 750–900 nm band-pass filter (light intensity: 300–750 mW/cm^2^) for 10 min sequentially. The temperature of the medium was monitored during irradiation using a thermal imaging camera (E6; FLIR, Wilsonville, OR, USA), which was between 50 and 55 °C by altering the light intensity.

### 2.4. Assessment of Anti-Cancer Drugs in In Vitro Cancer Cell Lines

Cells in a 24-well plate (3.0–10.0 × 10^4^ cells/mL/well) were treated with mitoxantrone dihydrochloride (MIT) (Tokyo Chemical Industry, Tokyo, Japan, #M3110) at 5 µM or *cis*-diammineplatinum (II) dichloride (CDDP) (Tokyo Chemical Industry, #D3371) at 100 µM for 1, 6, and 24 h.

### 2.5. Flow Cytometric Evaluation of Cell Death Morphology

The FM3A cells were washed once with 1 mL phosphate-buffered saline (PBS; Nissui Pharmaceutical, Tokyo, Japan), and then centrifuged at 1000× *g* for 3 min to remove the supernatant. The resulting pellets were suspended in Annexin binding buffer (Becton Dickinson, Franklin Lakes, NJ, USA, #556454) (2.0 × 10^6^ cells/mL) and filtered through a 48 µm nylon mesh (Tokyo Garasu Kikai, Tokyo, Japan) to obtain a single-cell suspension. Cells were then stained with DAPI (Thermo Fisher Scientific, Waltham, MA, USA, #62248) and AnnexinV-FITC (BioLegend, San Diego, CA, USA, #640906) to detect apoptotic and necrotic cell populations, respectively. The excitation and emission wavelengths were 405 nm and 450 nm for detecting DAPI and 488 nm and 530 nm for detecting Annexin V-FITC, using a FACSCanto™ II (Becton Dickinson). Analysis was performed using FlowJo™ (Becton Dickinson).

### 2.6. In Vitro Immunofluorescence Staining of HMGB1 and CRT

The FM3A cells were smeared onto glass slides using Smear Gell (Genostaff, Tokyo, Japan, #SG-01), and fixed with 4% paraformaldehyde (PFA; Nacalai Tesque, #09154-85) at room temperature for 10 min, then washed with PBS. Cells were treated with iFluor^®^ 488-WGA working solution (AAT Bioquest, Pleasanton, CA, USA, #25530) (2–10 µg/mL) for 10–30 min at room temperature and washed twice with Hank’s Buffer with HEPES. The cells were permeabilized with 0.5% Triton X-100 in PBS for 10 min at room temperature, followed by blocking with 1% bovine serum albumin (BSA; Nacalai Tesque, #01281-26) in PBS at room temperature for 1 h. Cells were stained with primary antibodies (Table S1) at 4 °C overnight, followed by incubation with secondary antibodies (Table S1) at room temperature for 1 h. Nuclei were stained with DAPI (1:5000, Thermo Fisher Scientific, #62248) with secondary antibodies and washed three times with PBS. Coverslips were mounted on slides using a ProLong Glass Antifade Mountant (cat# P36984, Invitrogen, Waltham, MA, USA). Images were captured using SLELLARIS 5 (Leica, Wetzlar, Germany). The cellular region was assigned as the region of interest (ROI) using the fluorescent image of the cell membrane with Cellpose 3.0 [15]. The intracellular area percentage of HMGB1 and intensity of CRT on the cell membrane were measured (Figure S9).

### 2.7. Preparation of Tumor-Bearing Mouse Model

C3H/He mice (5–6 weeks old, female) were purchased from Japan SLC (Shizuoka, Japan). All animals had ad libitum access to food and water and were allowed to acclimate to the animal facility for one week prior to the start of the experiments. Cancer cells were subcutaneously (s.c.) transplanted into syngeneic mice at a concentration of 1.0–3.5 × 10^6^ cells/100 µL of Hanks’ balanced salt solution (Gibco, Grand Island, NY, USA, #14025-092). Tumor volume was calculated using the formula: 1/2 × a × b2, where a and b represent the largest and smallest tumor diameters, respectively. Mice were euthanized by cervical dislocation when the tumor volume exceeded 2000 mm^3^, with the tumor volume and body weight recorded at that point as final data. All animal procedures reported in this study were approved by the Ethics Committee of Chiba university (CA 6-96), and conducted in accordance with Chiba university regulations for the implementation of animal experiments. All animal reporting was conducted in accordance with ARRIVE guidelines 2.0 [16].

### 2.8. Anti-Cancer Drug Treatment in Tumor-Bearing Mice

The mice received intravenous injections of mitoxantrone at 3 mg/kg twice, 42 h apart, or intraperitoneal injections of CDDP at 4 mg/kg three times, 2 days apart.

### 2.9. PTT of Tumor Tissues Using Gold Nanorods and NIR Irradiation

Gold nanorods (4 µg in 50 µL PBS) were administered subcutaneously to the tumor tissues. The tumors were then exposed to NIR light (750–900 nm) for 10 min, with the mice under isoflurane anesthesia. The temperature of the tumors was monitored using a thermal imaging camera (E6, FLIR), with the temperature controlled between 50 and 55 °C by adjusting the light intensity (300–750 mW/cm^2^).

### 2.10. In Vivo Immunofluorescence Staining

When the tumor size reached 100–200 mm^3^, the mice were treated with anti-cancer drugs or PTT, as described above. Tumors were harvested 3 days after the last anti-cancer drug treatment or 1 day after PTT. Tumors were then fixed in 4% PFA for 15 min at 4 °C. The tissues were transferred to 20% sucrose in PBS for 3 h, and subsequently incubated with 30% sucrose in PBS overnight at 4 °C. Finally, the tissues were embedded and frozen in optimal cutting temperature (OCT) compound (Sakura Finetek, Tokyo, Japan, #4583). Fixed tissues were sectioned at a thickness of 10 μm and mounted on glass slides (Matsunami glass, Osaka, Japan, SFRC-01). The slides were blocked with 1% BSA in PBS and stained with primary antibodies (Table S1) at 4 °C overnight, followed by treatment with fluorescence-conjugated secondary antibodies (Table S1) and DAPI (1:5000, Thermo Fisher Scientific, #62248) for 1 h at room temperature. After incubation, the coverslips were mounted on slides using ProLong Glass Antifade Mountant (Invitrogen, Waltham, MA, USA, #P36984). Images were captured using a BZ-X710 microscope (Keyence, Tokyo, Japan). Dead cells in tissues were detected using the terminal deoxynucleotidyl transferase-mediated dUTP nick-end labeling (TUNEL) assay.

### 2.11. PTT and aCTLA-4 mAb Combination Therapy

When tumors derived from FM3A cells reached a volume of 50–100 mm, the tumor-bearing mice were treated with a second round of PTT 4 days after the initial PTT. Animals were randomly assigned to treatment groups, and each animal served as an independent experimental unit. As part of ICI treatment, FM3A tumor-bearing mice received intraperitoneal injections of aCTLA-4 mAb (Table S1) at 50 µg/mouse (approximately 2.5 mg/kg) or isotype control IgG antibodies (Table S1) in 100 µL PBS on days 5, 8, and 12 (post-tumor inoculation). The tumor volume was measured as described above. On the last day, the tumor growth-inhibition (TGI) ratio was calculated using the formula: TGI = [1 − (tumor volume of the treatment group)/(tumor volume of the control group)] × 100 (%). The interaction index between the combination therapy and monotherapy was calculated according to a previously described method [17]. Drug combinations were classified based on the 95% confidence interval (CI) where combinations with CI less than zero were considered supra-additive; those with CI zero were considered additive, and those with CI greater than zero were considered sub-additive. Mice were sacrificed when the tumor volume exceeded 2000 mm^3^, with the tumor volume and body weight recorded at that point as final data. The sample size was chosen based on previous studies.

### 2.12. Evaluation of Gene Expression in Tumors

Tumors were harvested on day 14 after starting the combination therapy. Total RNA was extracted using RNAzol RT Reagent (Molecular Research Center, Cincinnati, OH, USA, #RN190) and Direct-zol RNA MiniPrep kit (Zymo Research, Irvine, CA, USA, #R2052), according to the manufacturer’s instructions. cDNA was synthesized using the ReverTra AceR qPCR RT Master Mix (Toyobo, Osaka, Japan). Reverse Transcription quantitative PCR (RT-qPCR) was performed using the ThunderBird SYBRR qPCR Mix (Toyobo) and StepOne system (Applied Biosystems, Waltham, MA, USA). Granzyme B and IFN-γ mRNA expression levels were normalized to those of GAPDH and analyzed using the 2^−ΔΔCt^ method. The following primers were used: mouse GAPDH forward 5′-CGACTTCAACAGCAACTCCCACTCTTCC-3′ and reverse 5′-TGGGTGGTCCAGGGTTTCTTACTCCTT-3′, mouse Granzyme B forward 5′-GGCCCACAACATCAAAGAAC-3′ and reverse 5′-GCAGCATGATGTCATTGGAG-3′, mouse IFN-γ forward 5′-CTTCTTCAGCAACAGCAAGG-3′ and reverse 5′-TGAGCTCATTGAATGCTTGG-3′.

### 2.13. Statistical Analysis

Data are presented as the mean value ± standard error (S.E.). Multiple comparisons were conducted using one-way ANOVA with Tukey’s test. *p* values (both sides) were considered significant if <0.05. Statistical analyses were performed using GraphPad Prism 10.4.2 (GraphPad software, Boston, MA, USA).

## 3. Results

### 3.1. Distinct Cell Death Morphology Induced by PTT and Anti-Cancer Drugs

**Figure 1:**
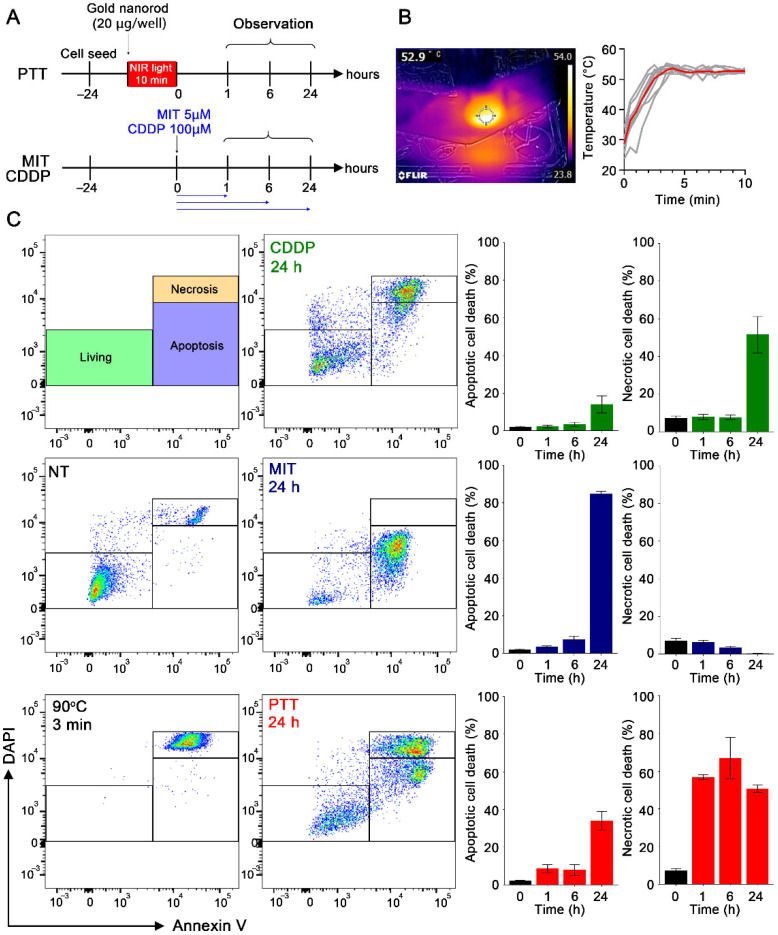
In vitro viability and cell death induced by MIT, CDDP, and PTT. (**A**) Experiment schedule for in vitro photothermal therapy (PTT) using gold nanorods and near-infrared (NIR) light. The temperature of the culture medium was maintained at 50–55 °C by adjusting the NIR light intensity after adding gold nanorods. Experiment schedule for in vitro mitoxantrone (MIT) and *cis*-diammineplatinum (II) dichloride (CDDP) treatment. (**B**) Representative thermal camera image during PTT and the temperature of the culture medium. (**C**) Density plot of Annexin V-DAPI method after each treatment and the analysis of cell death morphology. The red, blue, and green bars represent cell death induced by PTT, MIT, and CDDP, respectively. Data are presented as mean ± S.E.; *n* = 3.

FM3A cancer cells were treated with either PTT using gold nanorods and NIR irradiation (Figure S1), or anti-cancer drugs (Figure 1A). For PTT, the temperature of the medium was maintained at 50 °C for at least 7 min (Figure 1B). Cells were treated with anti-cancer drugs at concentrations, which can damage cancer cells sufficiently (Figure S2). After single cells were selected (Figure S3), we evaluated the ratio of apoptotic cell death to necrotic cell death. Cells heated at 90 °C for 3 min were used as the positive necrotic cell control. Cell death morphology was determined as follows: Cells heated at 90 °C for 3 min, which showed positive results in Annexin V staining and strongly positive results in DAPI staining, were used as a positive control for accidental necrotic cells, indicative of rapid physicochemical-induced cell death. In addition, we defined annexin V-positive and DAPI-negative or weakly positive cells as apoptotic cells because this population tended to increase over time, indicative of programmed cell death (Figure 1C and Figure S4). CDDP primarily induced necrotic cell death with limited apoptotic cell death, while MIT primarily triggered apoptotic cell death with little to no necrosis. In contrast, PTT-treated FM3A cells showed a rapid increase in necrotic cells with increased membrane permeability, as well as a gradual increase in apoptotic cells over a period of 24 h. These results demonstrate that while PTT and anti-cancer drugs can effectively induce FM3A cell death, the morphology and time course of cell death induced by MIT, CDDP, and PTT differ.

### 3.2. Extracellular Release of HMGB1 Induced by PTT and Anti-Cancer Drugs

**Figure 2:**
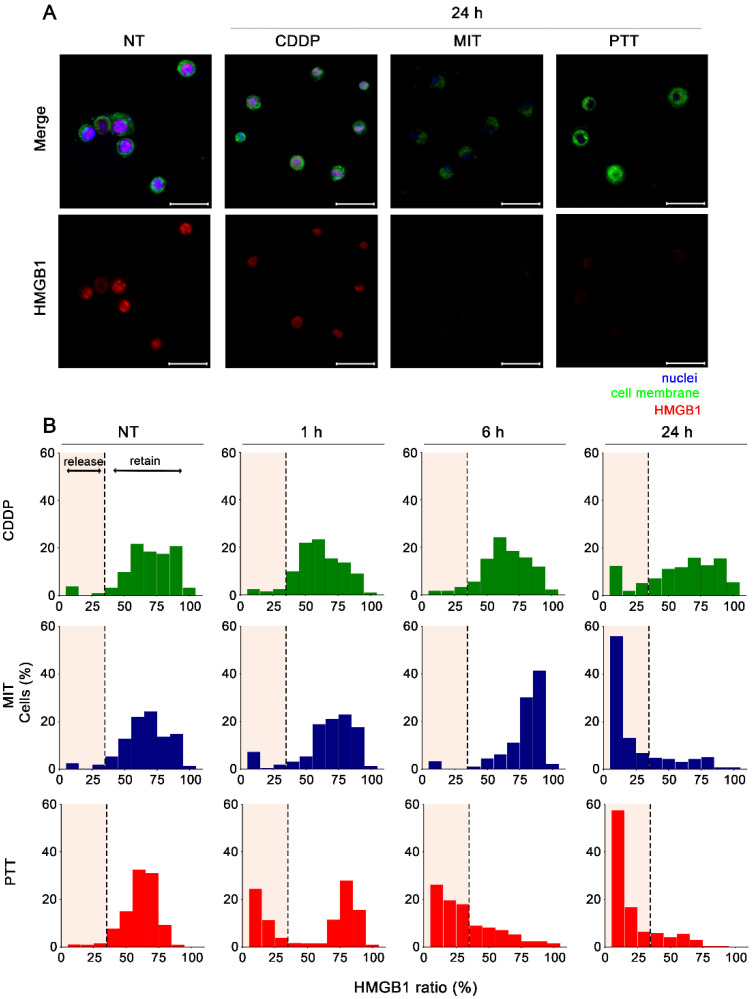
In vitro evaluation of HMGB1 extracellular release. (**A**) To evaluate the HMGB1, intracellular HMGB1 was visualized. FM3A cells were treated with PTT at 50–55 °C for 10 min, MIT (5 µM), or CDDP (100 µM), followed by incubation for 1, 6, or 24 h. Cells were fixed, permeabilized, and stained with anti-HMGB1 (red) and DAPI (blue). Scale bar: 30 µm. (**B**) The region surrounded by the stained cell membrane was considered as the internal area of the cell, which was assigned as the region of interest (ROI). The percentage of HMGB1-positive area within the ROI was calculated for each cell. Cells with an HMGB1 area lower than 30% were designated as HMGB1 releasing. Data are representative of three independent biological replicates (*n* = 177–249 cells from seven images).

Given that the extracellular release of DAMPs such as HMGB1 is a hallmark of ICD [6,18], we compared the ICD induction ability of PTT and anti-cancer drugs by observing the localization of HMGB1 before and after treatment in FM3A cells (Figure 2A and Figure S5). As the intracellular area percentage of the HMGB1 signal in non-treated cells ranged between 30 and 80%, we originally defined cells whose HMGB1 area percentage was ≥30% as ‘HMGB1 retaining,’ while those ≤ 30% as ‘HMGB1 releasing.’ Although CDDP, a non-ICD inducer, sufficiently induced cell death (Figure 1C), the CDDP-induced extracellular release of HMGB1 was limited. In contrast, MIT, an ICD inducer [19,20,21], induced HMGB1 release in approximately 80% of cells. HMGB1 release from PTT-treated cells was detected after 1 h of treatment, with over 80% of the cells showing HMGB1 release by 24 h (Figure 2B). Also, HMGB1 release by PTT and MIT was reproduced in MC38 cells (Figure S7). Taken together, the data suggests that the extent of HMGB1 release correlates with the ICD induction capacity of PTT and MIT.

### 3.3. CRT Cell Membrane Expression Induced by PTT and Anti-Cancer Drugs

**Figure 3:**
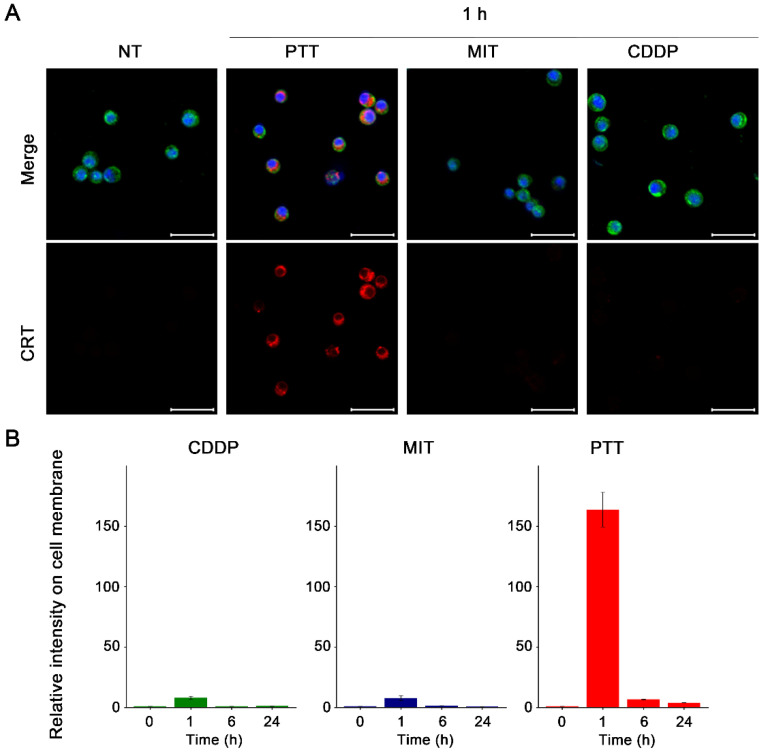
In vitro evaluation of CRT expression and localization. (**A**) To quantify the CRT expression on the cell membrane, the CRT on the cell membrane was visualized. FM3A cells were treated with PTT at 50–55 °C for 10 min, or MIT (5 µM), or CDDP (100 µM), followed by incubation for 1, 6, or 24 h. Cells were fixed, permeabilized, and stained with anti-CRT (red) and WGA (green) DAPI (blue). Scale bar: 30 µm. (**B**) The region surrounded by the stained cell membrane was considered as the internal area of the cell, which was assigned as ROI. The fluorescence intensity of CRT on the cell membrane in the ROI was measured for each cell. Data are presented as mean ± S.E. of three independent biological replicates (*n* = 274–816 cells from seven images).

To further investigate the ICD profile induced by PTT and anti-cancer drugs, we examined the expression and localization of CRT before and after treatment in FM3A cells (Figure 3A and Figure S6). CRT is a molecular chaperone in the endoplasmic reticulum. During ICD of cancer cells, CRT translocates from the endoplasmic reticulum to the cell membrane, where it acts as an “eat-me” signal to facilitate phagocytosis [22]. Because the expression of CRT on the cell membrane plays a key role in phagocyte recognition, the mean fluorescence intensity (MFI) of CRT co-localized with the cell membrane was calculated. CDDP did not alter CRT expression or localization. Notably, in contrast to our expectations, MIT also did not alter the expression or localization of CRT. However, CRT expression in PTT-treated cells increased drastically and was translocated to the cell membrane (Figure 3B). PTT efficiently increased the CRT expression on cell membrane in MC38 cells, too (Figure S8).

### 3.4. DAMPs Release in Tumor Tissues Following Treatment with PTT and Anti-Cancer Drugs

**Figure 4:**
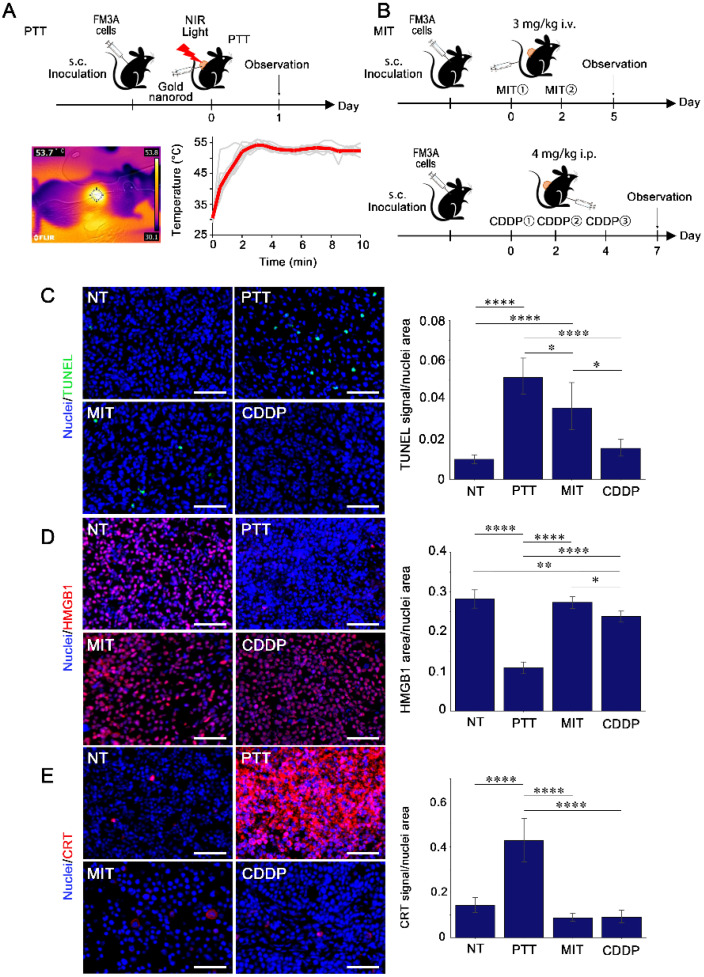
In vivo evaluation of cell death and DAMPs after treatment with PTT and anti-cancer drugs. (**A**) Experiment schedule for PTT treatment to tumor-bearing mice, representative thermal camera image during PTT, and the temperature on the surface of the tumor. (**B**) Experiment schedule for anti-cancer drug treatment to tumor-bearing mice. (**C**) Terminal deoxynucleotidyl transferase-mediated dUTP nick-end labeling (TUNEL) staining of dead cells in FM3A tumor tissues after each treatment (*n* = 154–327 images from three biological replicates per group). The blue and green signals represent nuclei and TUNEL, respectively. Scale bar: 50 µm. The right graph shows the number of TUNEL signals normalized by the nuclei area. (**D**,**E**) Immunofluorescence staining of HMGB1 and CRT in FM3A tumor tissues after each treatment (*n* = 218–654 images from three biological replicates per group). The blue and red signals represent nuclei and HMGB1 or CRT, respectively. Scale bar: 50 µm. The right graph shows the HMGB1 area or CRT signal normalized by the nuclei area. ^∗^ *p* < 0.05, ^∗∗^ *p* < 0.01, ^∗∗∗∗^ *p* < 0.0001 (one-way ANOVA followed by Tukey’s test).

We demonstrated that PTT efficiently induced ICD-related cellular responses in vitro in FM3A and MC38 cells. Additionally, we observed cell death and DAMPs release in the FM3A tumor tissues after each treatment (Figure 4A,B). To induce cell death in tumor tissues for ICD evaluation, MIT and CDDP were administered at the maximum tolerated dose without inducing toxicity (Figure 4B). The dosage of the anti-cancer drug was determined as the highest dose immediately below the threshold of observed toxicity, based on previous reports [23,24] and a preliminary experiment. After treatment, cell death in the tumors was detected using the TUNEL assay [25]. Even after treatment with the maximum tolerated dose, cell death was not detected following CDDP treatment. In contrast, the number of TUNEL-positive cells increased markedly after treatment with PTT or MIT (Figure 4C). Moreover, we observed the localization of HMGB1 and CRT. CDDP had little effect on HMGB1 release and CRT expression in tumor tissues (Figure 4D,E), potentially attributed to CDDP-induced inefficient cell death. Although cell death occurred in the tumor tissues after treatment with MIT, neither HMGB1 release nor CRT expression was observed. Treatment with PTT induced remarkable HMGB1 release and increased the expression of CRT. These results demonstrate that PTT can efficiently induce ICD-related signaling in tumor tissues and could be a promising combination therapy for cancer immunotherapy.

### 3.5. Combination Therapy with PTT and ICIs

**Figure 5:**
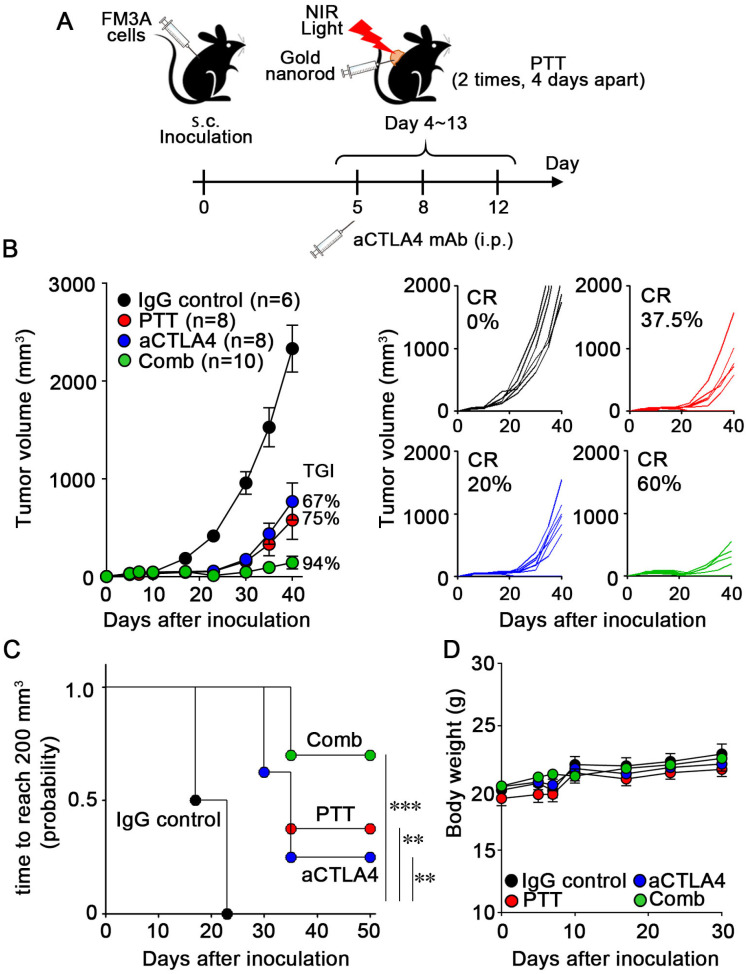
Combination therapy with PTT and an immune checkpoint inhibitor. (**A**) FM3A tumor-bearing mice were treated with either PTT, aCTLA-4 mAb, or their combination. PTT was performed as described above. Mice were treated with aCTLA-4 mAbs at 50 µg/mouse or isotype control IgG antibodies on days 5, 8, and 12 after inoculation. (**B**) Average and individual volume of FM3A tumors. (**C**) Time to progression based on a predefined tumor volume threshold (200 mm^3^) and the average body weight of FM3A tumor-bearing mice after treatment. Log-rank test with Bonferroni correction was used to assess survival differences (^∗∗^ *p* < 0.01, ^∗∗∗^ *p* < 0.001). (**D**) The body weight of mice during treatment. Data are presented as mean ± S.E. TGI, tumor growth inhibition; CR, complete response.

We hypothesized that PTT treatment could promote the cancer-immunity cycle, which in turn may accelerate the immune response to FM3A cells [26]. Therefore, we explored whether ICD-associated hallmarks induced by PTT are sufficient to enhance responsiveness to immune checkpoint blockade, rather than to directly compare the therapeutic efficacy of PTT with that of ICD-associated chemotherapeutic agents in vivo. We hypothesized that ICD induction by PTT could further promote the priming phase activation, which occurs immediately after ICD, thereby improving the therapeutic efficacy of aCTLA-4 mAb treatment (Figure 5A). Single treatment with either aCTLA-4 mAb or PTT suppressed FM3A tumor growth, while the combination therapy showed better anti-tumor effects, with TGI and CR of 94% and 60%, respectively (Figure 5B). As tumors tend to exhibit aggressive proliferation once their volume exceeds 200 mm^3^, we defined the duration during which the tumor volume remained less than 200 mm^3^ as progression-related readout. The combination of PTT with aCTLA-4 mAb also improved progression-related readout compared to the single treatment, without any weight loss during the observation period (Figure 5C,D). We also assessed the interaction index between combination and monotherapies [17]. The interaction index of the combination therapy was found to be −0.283 (95% CI, −0.362 to −0.204), which indicates a supra-additive effect of PTT and aCTLA-4 mAb relative to monotherapies. These results suggest that PTT and blockade of CTLA-4 could synergistically enhance the immune response in FM3A cells.

### 3.6. Conversion of TME from Cold to Hot by PTT

**Figure 6:**
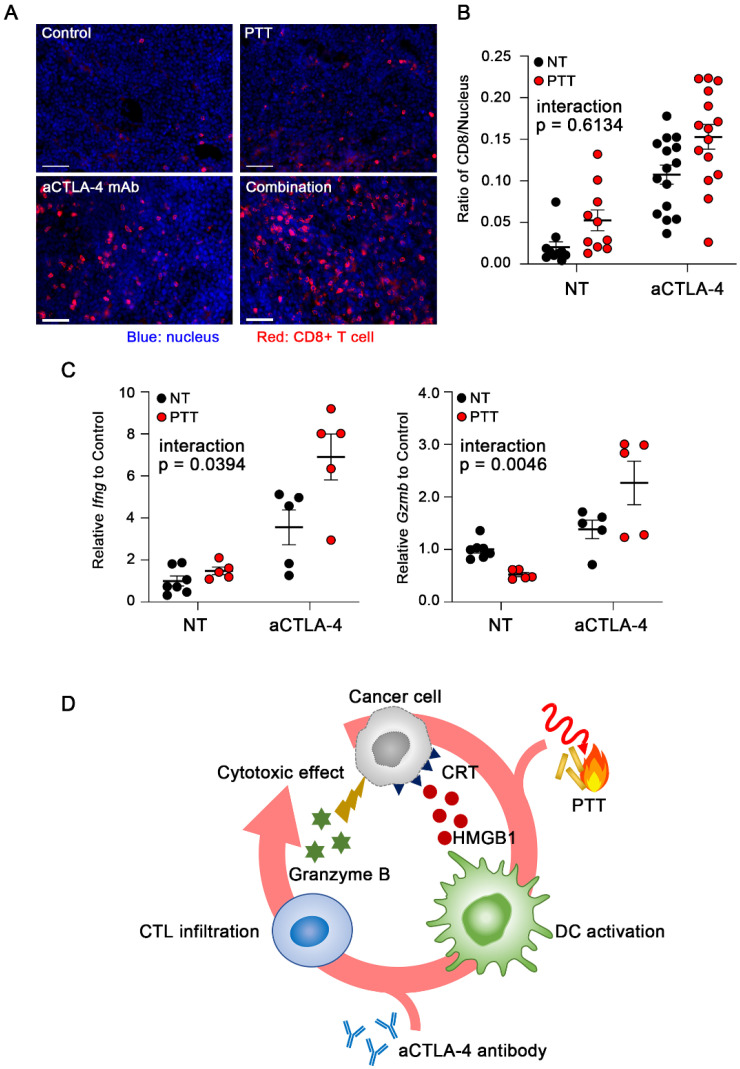
PTT combined with aCTLA-4 converted the TME to an immunologically hot state. (**A**) Immunofluorescence staining of CD8^+^ T cells in FM3A tissues after treatment with either PTT, aCTLA-4 mAb, or their combination. The blue and red signals represent nuclei and CD8^+^ T cells, respectively. Scale bar: 100 μm. (**B**) Relative area of CD8^+^ T cells to that of the nucleus was calculated (10–15 images per group). Two-way ANOVA showed significant main effects of aCTLA-4 mAb treatment (*p* < 0.0001) and PTT (*p* = 0.0042), with no interaction. (**C**) Gene expressions of IFN-γ and granzyme B in FM3A tumors treated with either PTT, aCTLA-4 mAb, or their combination (*n* = 5–7). Two-way ANOVA revealed a significant interaction between aCTLA-4 mAb and PTT. (**D**) The schematic diagram of a possible mechanism for the synergistic effect achieved by the combination of PTT with ICI through the cancer-immunity cycle.

We investigated whether the enhanced anti-tumor effect of the combination therapy (PTT + aCTLA-4 mAb) was mediated through the transformation of cold tumors into hot tumors. The number of infiltrating CD8^+^ T cells in FM3A tumor tissue was elevated by treatment with either PTT or aCTLA-4 mAb, which was further increased by the combination treatment (Figure 6A,B). The activation of CD8^+^ T cells in tumors was assessed by evaluating the expression of INF-γ and granzyme B [27]. The combination treatment showed significantly enhanced expressions of both *Ifng* and *Gzmb* as compared to treatment with either PTT or aCTLA-4 mAb alone (Figure 6C). These results demonstrate that the combination of PTT and aCTLA-4 mAb could convert cold tumors into hot tumors.

## 4. Discussion

In immunotherapy, the induction of ICD is a promising strategy, as it promotes the cancer-immunity cycle by enhancing immune cell recognition and activation [5]. Although several therapeutic modalities are known to induce ICD, few studies have addressed the differences in ICD profiles induced by these modalities. In this study, we demonstrated that PTT is a superior therapeutic modality compared to anti-cancer drugs in terms of ICD induction process, based on investigations using a murine tumor model.

The therapeutic efficacy of PTT depends on the temperature and duration of heating (i.e., thermal dose) [28,29]. Numerous studies have shown that therapeutic effects are obtained by maintaining tumor temperatures at approximately 50 °C for 5–10 min [14,30,31,32,33]. Additionally, maintaining tumor temperatures above 43 °C during PTT is sufficient to exert anti-tumor effects [34]. In the present study, in vitro cancer cells were efficiently killed by applying a thermal dose of 50 °C for 10 min using gold nanorods and NIR irradiation (Figure 1). However, prolonged irradiation of in vivo tumor tissues at 50–55 °C for more than 10 min may lead to tissue damage, including burns to the adjacent normal tissues, making it practically difficult to further increase the thermal dose. The observed immunogenic responses under specific thermal conditions should not be interpreted as supporting the general superiority of PTT across all temperature ranges. Evaluation of lower thermal doses and detailed temperature–response relationships is an important topic from the perspective of translation to clinical practice.

To detect ICD-related signaling, we assessed the translocation of HMGB1 from the nucleus to the extracellular space and CRT from the endoplasmic reticulum to the cell surface using an image-based evaluation. This is because HMGB1 and CRT exert immunological activity only after redistribution from their native intracellular compartments.

HMGB1 released into the extracellular space is recognized by Toll-like receptor 4 (TLR-4)-expressing immune cells such as macrophages and dendritic cells [35,36], activating the priming phase and subsequent steps in the cancer-immunity cycle. Although cell membrane permeability is considered an important factor for the extracellular release of HMGB1 [37,38], our results showed no such correlation (Figure 1C and Figure 2). This implies that apart from cell membrane permeability, other factors, such as nuclear-to-cytoplasmic translocation, are also essential for the extracellular release of HMGB1.

CRT acts as an ‘eat-me’ signal that promotes phagocytosis by antigen-presenting cells, such as dendritic cells [39]. We observed that PTT not only induced CRT translocation to the plasma membrane but also markedly increased its expression (Figure 3). This result is consistent with previous reports that the expression of CRT, which belongs to the heat shock protein family, is strongly upregulated by heat shock [40,41]. While anthracycline-based anti-cancer drugs such as mitoxantrone generally promote CRT translocation to the plasma membrane via endoplasmic reticulum stress [19], this was not observed in our study (Figure 3). The divergent responses between MIT and PTT may reflect the modality-specific stress profiles ascribed to exposure dynamics. These results highlight the advantage of PTT in promoting CRT expression and its translocation to the cell membrane under the conditions examined, whereas anthracycline drugs did not elicit comparable calreticulin surface exposure within the experimental time frame of this study.

The efficient induction of ICD in tumor tissues is also important for improving therapeutic efficacy. In our tumor-bearing mouse model, anti-cancer drugs failed to induce cell death, HMGB1 release, or increase CRT expression as effectively as PTT (Figure 4C–E). Systemically administered chemotherapeutic agents do not necessarily achieve sufficient exposure within tumor tissue to induce ICD-associated responses [42], likely due to limited tumor penetration and insufficient local drug availability. Because intratumoral drug levels were not directly assessed in this study, inadequate exposure within the tumor may explain the absence of detectable HMGB1 redistribution and CRT surface exposure in vivo, even in the presence of TUNEL-positive tumor cell death. Also, although the in vitro induction of HMGB1 extracellular release by MIT is fully consistent with findings from previous studies, the contradiction that HMGB1 release was not detected despite MIT inducing DNA damage–associated cell death in vivo suggests that ICD is likely determined not only by a drug’s cytotoxic capacity but also by its spatial distribution and concentration within the tumor. These results support the idea that comprehensive research on ICD necessitates evaluation within the in vivo environment. In contrast, localized PTT directly delivers physical energy to tumor tissue, potentially overcoming such delivery-related constraints and thereby enabling more consistent induction of ICD-associated signaling in vivo.

ICIs are widely used in clinical immunotherapy for cancer treatment. In this study, the complete response rate increased from 37.5% to 60% when ICIs were combined with PTT (Figure 5B). The observed increase in CD8^+^ T cell infiltration in tumor tissues, as well as increased expression of INF-γ and granzyme B, suggests that PTT-induced ICD-related cellular responses promoted the activation of dendritic cells, enhanced antigen presentation to T cells, and increased CTL infiltration into the tumor. These immune responses may have normalized the immune system balance—such as the effector-to-regulatory T cell ratio—resulting in a more immunologically hot TME and an improved ICI response rate (Figure 6). Furthermore, during the period observed in this study, the combination therapy substantially increased the therapeutic effects compared to ICI or PTT alone, highlighting the advantage of combining PTT with immunotherapy through immune sensitization to ICI. Overall survival and durable complete tumor regression would illustrate the long-term outcome.

CRT exposure and HMGB1 release following PTT, as well as downstream immune activation in vivo, including increased CD8^+^ T cell infiltration and enhanced therapeutic efficacy when combined with ICIs, support the conclusion that PTT elicits coordinated immunogenic signaling.

PTT, as a minimally invasive and selective cancer therapy, is gaining attention due to its ability to apply localized thermal stress specifically to tumor tissues. This study provides insights into the ICD profiles induced by different therapeutic modalities and highlights the potential of PTT as an anti-cancer drug-free approach to be used in combination with immunotherapy. Despite the promising therapeutic efficacy observed in this study, several challenges remain for clinical translation. These include the relatively limited tumor accumulation efficiency of gold nanorods following systemic administration, heterogeneous biodistribution across organs, and uncertainties regarding long-term safety and clearance. In particular, variability in enhanced permeability and retention (EPR) effects among patients may reduce tumor-specific accumulation [43]. Furthermore, gold nanorods may be captured in reticuloendothelial organs such as the liver and spleen, raising concerns about long-term toxicity and biocompatibility [44]. Future studies focusing on active targeting strategies, comprehensive long-term toxicity evaluations, and thorough investigation of physicochemical properties, such as surface PEG density, will be essential to facilitate clinical translation.

This study has some limitations. First, our investigation focused on two major DAMPs—HMGB1 and CRT; however, a more comprehensive analysis of DAMPs release would be valuable because the activation of antitumor immunity involves a complex interplay of signals. Second, we measured tumor surface temperature using a thermal imaging camera; therefore, the temperature deep within the tumor and the variability in heat distribution within the tumor remain debatable. Future studies employing methods for accurately measuring intra-tumoral temperatures [45,46] and predicting the thermophysical properties of tumor tissues [47] will further clarify these issues.

Third, the tumor-bearing mouse model used in this study is known to be immunologically active and widely used for immunotherapy studies [48,49]; therefore, validation in other models with different tumor immune microenvironments is essential to generalize our conclusions. Moreover, the limited induction of ICD-related signaling by anti-cancer drugs in in vivo may reflect limited effective drug exposure within tumor tissue, rather than a lack of intrinsic immunogenic potential.

In addition, the experimental conditions were limited, with MIT examined at a single concentration in a small number of cancer cell lines. Accordingly, MIT was used as a reference compound under these conditions, and the applicability of the present findings to other ICD-inducing drugs or tumor types should be interpreted with caution. Therefore, caution is required when extrapolating these results to other therapeutic settings, given the fundamental differences among treatment modalities in delivery route, mode of cell death induction, and exposure conditions. Comparative analyses with other treatment modalities, such as cryoablation, laser ablation, and radiotherapy, would provide valuable complementary perspectives.

Finally, functional validation of effector T cell responses—such as cytokine production, cytotoxic activity, and the induction of immunological memory—was not performed in this study. Such analyses will be critical for clarifying the relationship between immune activation, immune sensitization, and responsiveness to ICIs.

## 5. Conclusions

In conclusion, PTT induces ICD-associated signaling more efficiently than conventional anti-cancer drugs and holds promise to be a valuable combination therapy with immunotherapy, including ICIs. When selecting a combination therapy with ICI, it is important to observe the process of the anti-tumor immune activation in detail, including observation of cell death morphology, release of DAMPs, and the immune microenvironment of the tumor.

## Data Availability

The data presented in this study are available on request.

## Supplementary Materials

The following supporting information can be downloaded at: https://www.mdpi.com/article/10.3390/cancers18020287/s1, Table S1. List of antibodies used in this study; Figure S1. Characterization of PEG-modified gold nanorods; Figure S2. Concentration-dependent cytotoxicity of anti-cancer drugs; Figure S3. Concentration-dependent cytotoxicity of anti-cancer drugs; Figure S4. In vitro cell death induced by MIT, CDDP, and PTT; Figure S5. In vitro immunofluorescence of HMGB1; Figure S6. In vitro immunofluorescence of CRT; Figure S7. In vitro immunofluorescence of HMGB1; Figure S8. In vitro immunofluorescence of CRT; Figure S9. Method for quantification of ICD induction.
