# Supplementary material for: Photothermal Therapy-Induced Immunogenic Cell Death Synergistically Enhances the Therapeutic Effect of Immune Checkpoint Inhibitors
Source: Cancers (Basel). 2026 Jan 16;18(2):287. doi: 10.3390/cancers18020287 (PMC12838917; doi:10.3390/cancers18020287)
Supplement: Supplementary file 1 [file cancers-18-00287-s001.zip › cancers-4020517-supplementary.pdf]

Supplemental Information

Supplemental materials and methods

WST assay

To measure cell viability, 2-(2-methoxy-4-nitrophenyl)-3-(4-nitrophenyl)-5-(2, 4-disulfophenyl)-2H-tetrazolium monosodium salt (WST-8; Nacalai Tesque, Kyoto, Japan; #07553-15) was added to the culture medium. After 1 h of incubation, the absorbance of WST-8 formazan was measured using a FilterMax F5 (Molecular Devices, Sunnyvale, California, USA). The absorbance was determined at 450 nm against a reference wavelength of 620 nm. Curve fitting was performed using Python (version 3.12.3).

Characterization of AuNRs

After functionalized with thiol-terminated polyethylene glycol (PEG–SH), the morphology and size of AuNRs were observed by transmission electron microscopy (TEM; JEM-2100F, JEOL, Japan) operated at 80 kV. Optical absorption spectra were recorded using a UV–vis–NIR spectrophotometer (V-670, JASCO, Tokyo, Japan). The zeta potential of AuNRs was measured with a Zetasizer (Zetasizer Nano ZS, Malvern Instruments Ltd., Malvern, UK).

Supplemental Table

Table S1. List of antibodies used in this study.

Antibody	Conjugate	Host	Reference	Purpose	Dose of Dilution
anti-CTLA-4	-	Mouse	9D9, Bioxcell	Treatment	50 µg/mouse
control IgG	-	Mouse	MCP-11, Bioxcell		50 µg/mouse
anti-CRT	-	Rabbit	12228T, CST	IF	1/1000
anti-HMGB1	-	Rabbit	ab79823, abcam		1/1000
anti-rabbit IgG	Alexa647	Donkey	A31573, invitrogen		1/2000

IF, immunofluorescence.

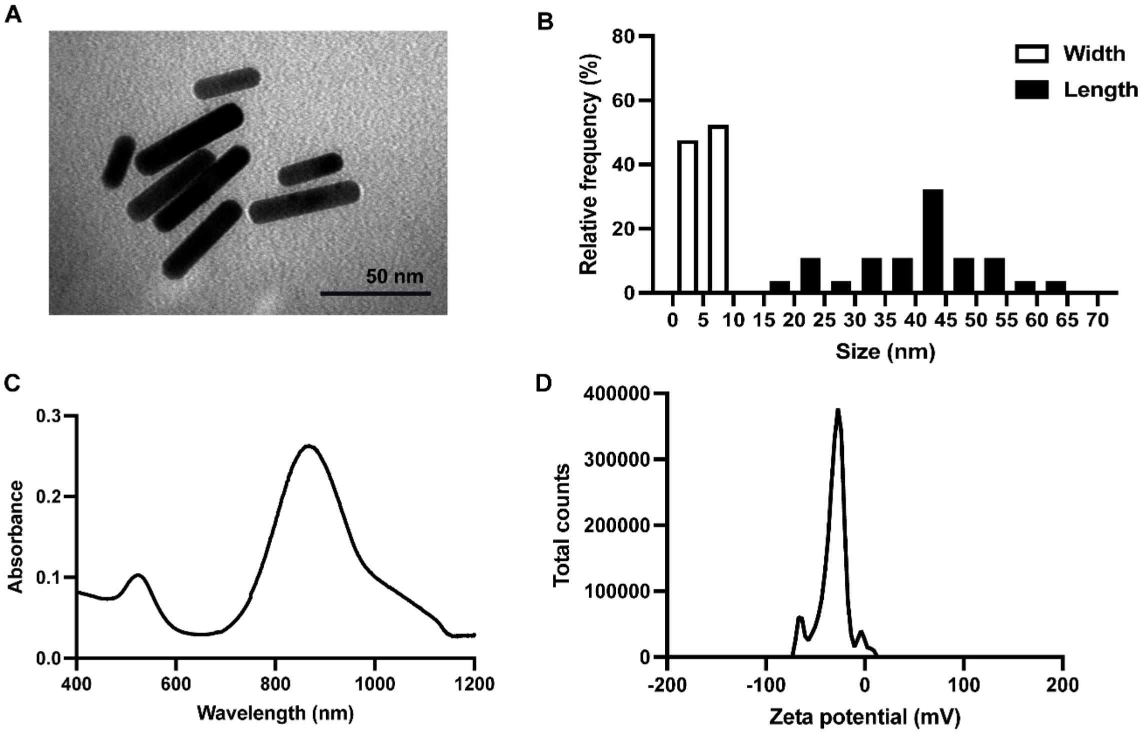

Figure S1. Characterization of PEG-modified gold nanorods.

(**A**) Representative TEM image of AuNRs. (**B**) Size distribution of AuNRs calculated from TEM images. (**C**) UV–vis–NIR absorption spectrum of AuNRs. (**D**) Zeta potential of AuNRs.

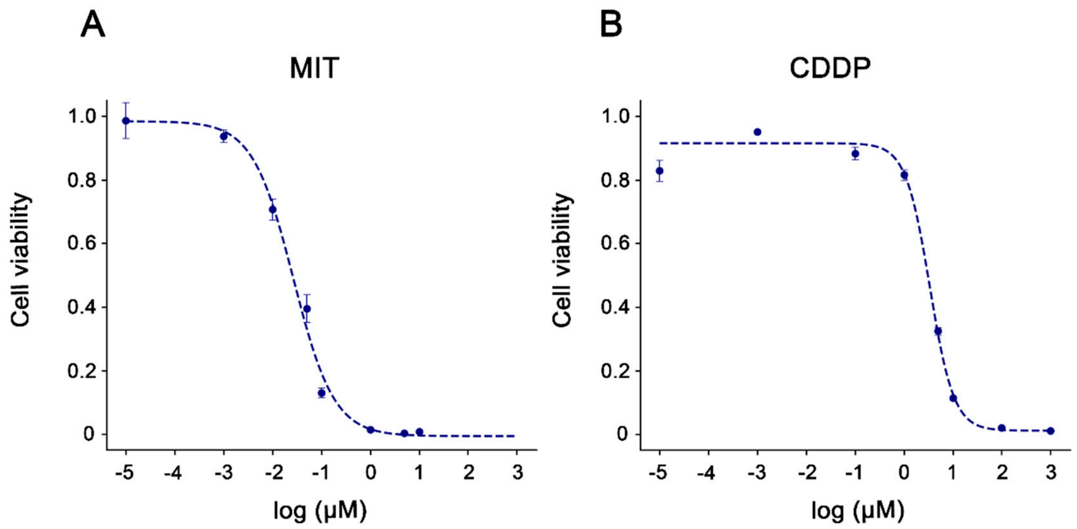

Figure S2. Concentration-dependent cytotoxicity of anti-cancer drugs

WST assay was conducted to measure the cytotoxic effect of (**A**) MIT or (**B**) CDDP on FM3A cancer cells. Data are presented as mean ± S.E.; n = 3. IC_{50} values of MIT and CDDP in FM3A cells were predicted as 2.6 × 10^{-2} μM and 3.1 μM.

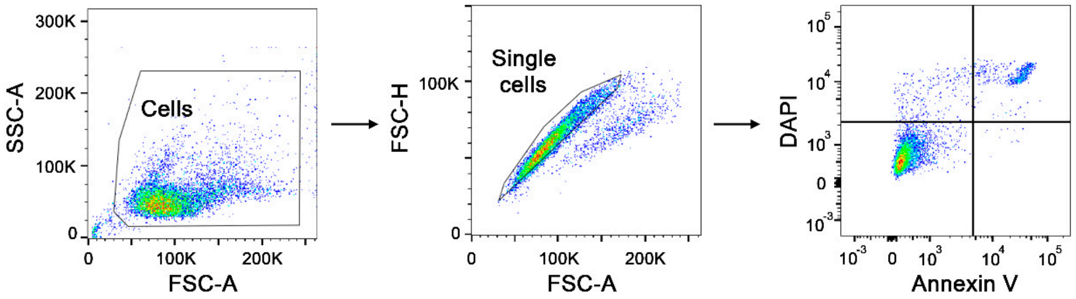

Figure S3. Concentration-dependent cytotoxicity of anti-cancer drugs

Gating strategy for cell death analysis. Cells were gated in an SSC-A and FSC-A dot plot to select cells and then gated in an FSC-H and FSC-A dot plot to eliminate doublets. The singlet gate was further analyzed for the Annexin V and DAPI intensity

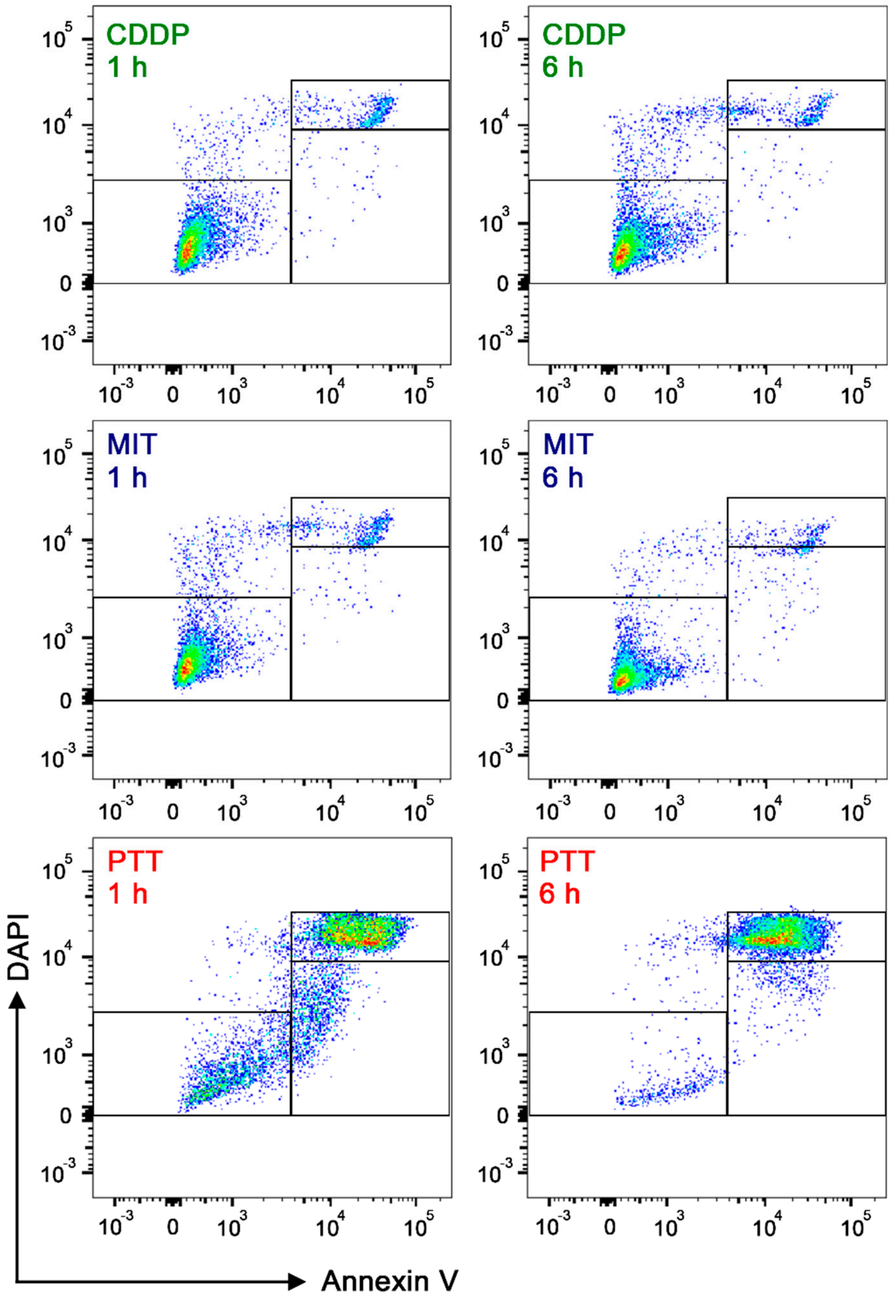

Figure S4. In vitro cell death induced by MIT, CDDP, and PTT

FM3A cells were treated with PTT at 50–55°C for 10 min, or MIT (5 μM), or CDDP (100 μM), followed by incubation for 1, 6, or 24 h. Cell death morphology was analyzed using flow cytometry.

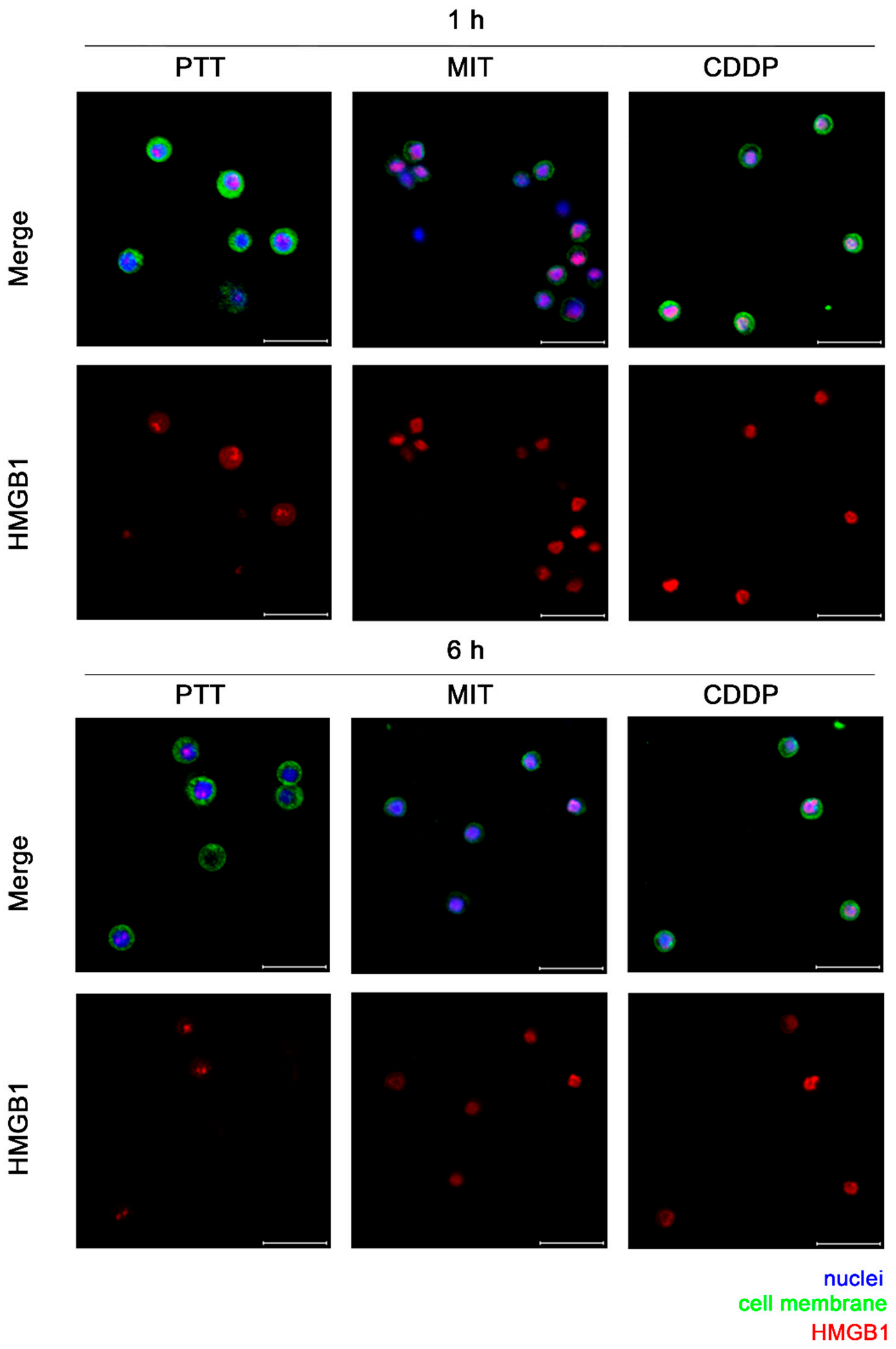

Figure S5. In vitro immunofluorescence of HMGB1

To evaluate the extracellular release of high mobility group box 1 (HMGB1), intracellular HMGB1 was visualized. FM3A cells were treated with PTT at 50–55°C for 10 min, or MIT (5 μM), or CDDP (100 μM), followed by incubation for 1, 6, or 24 h. Cells were fixed, permeabilized, and stained with anti-HMGB1 (Red) and DAPI (Blue). Scale bar: 30 μm.

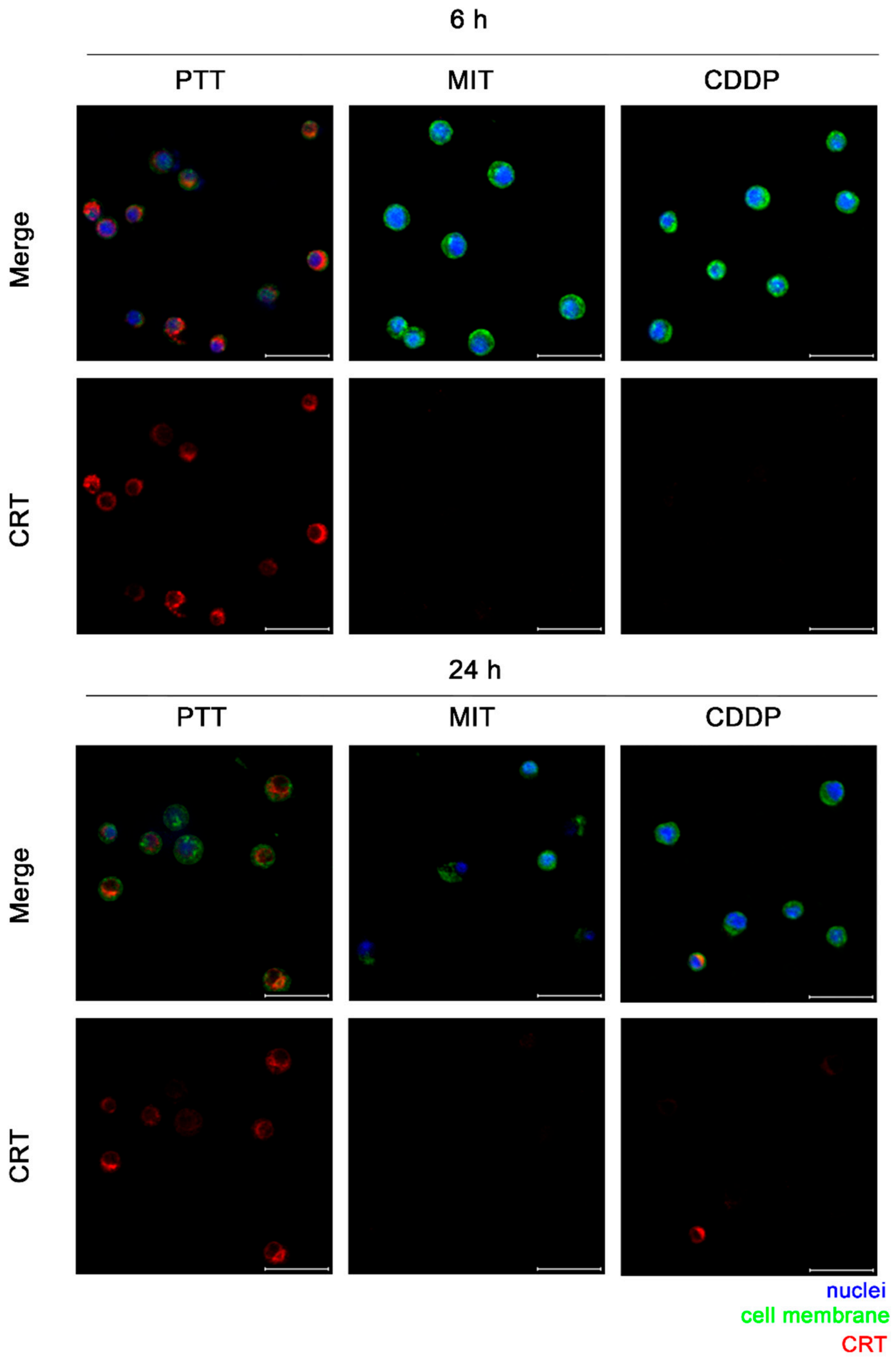

Figure S6. In vitro immunofluorescence of CRT

To quantify the calreticulin (CRT) expression on the cell membrane, the CRT on the cell membrane was visualized. FM3A cells were treated with PTT at 50–55°C for 10 min, or MIT (5 μM), or CDDP (100 μM), followed by incubation for 1, 6, or 24 h. Cells were fixed, permeabilized, and stained with anti-CRT (Red) and DAPI (Blue). Scale bar: 30 μm.

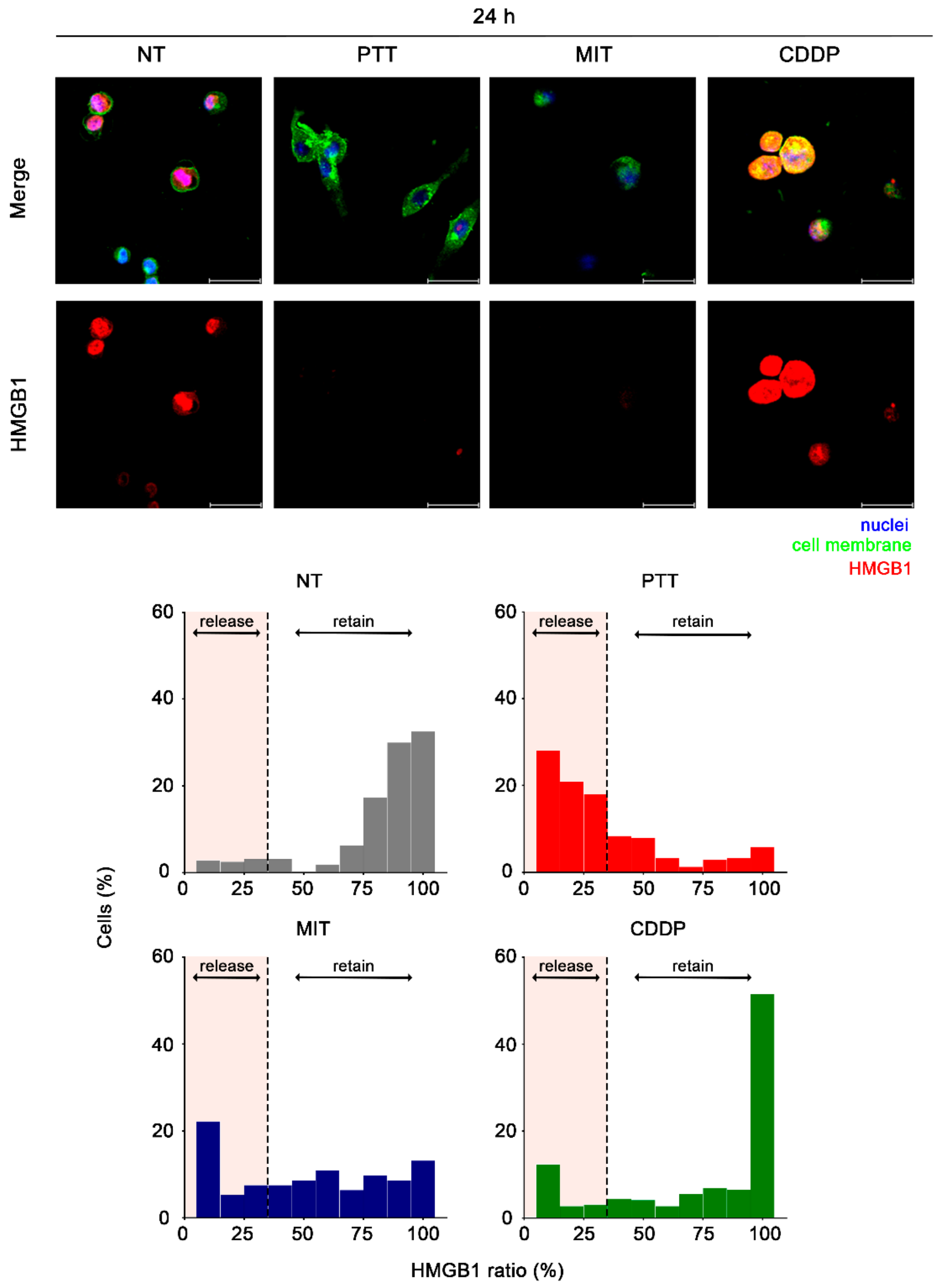

Figure S7. In vitro immunofluorescence of HMGB1

To evaluate the extracellular release of high mobility group box 1 (HMGB1), intracellular HMGB1 was visualized. MC38 cells were treated with PTT at 50–55°C for 10 min, or MIT (5 μM), or CDDP (100 μM), followed by incubation 24 h. Cells were fixed, permeabilized, and stained with anti-HMGB1 (Red) and DAPI (Blue). Scale bar: 30 μm.

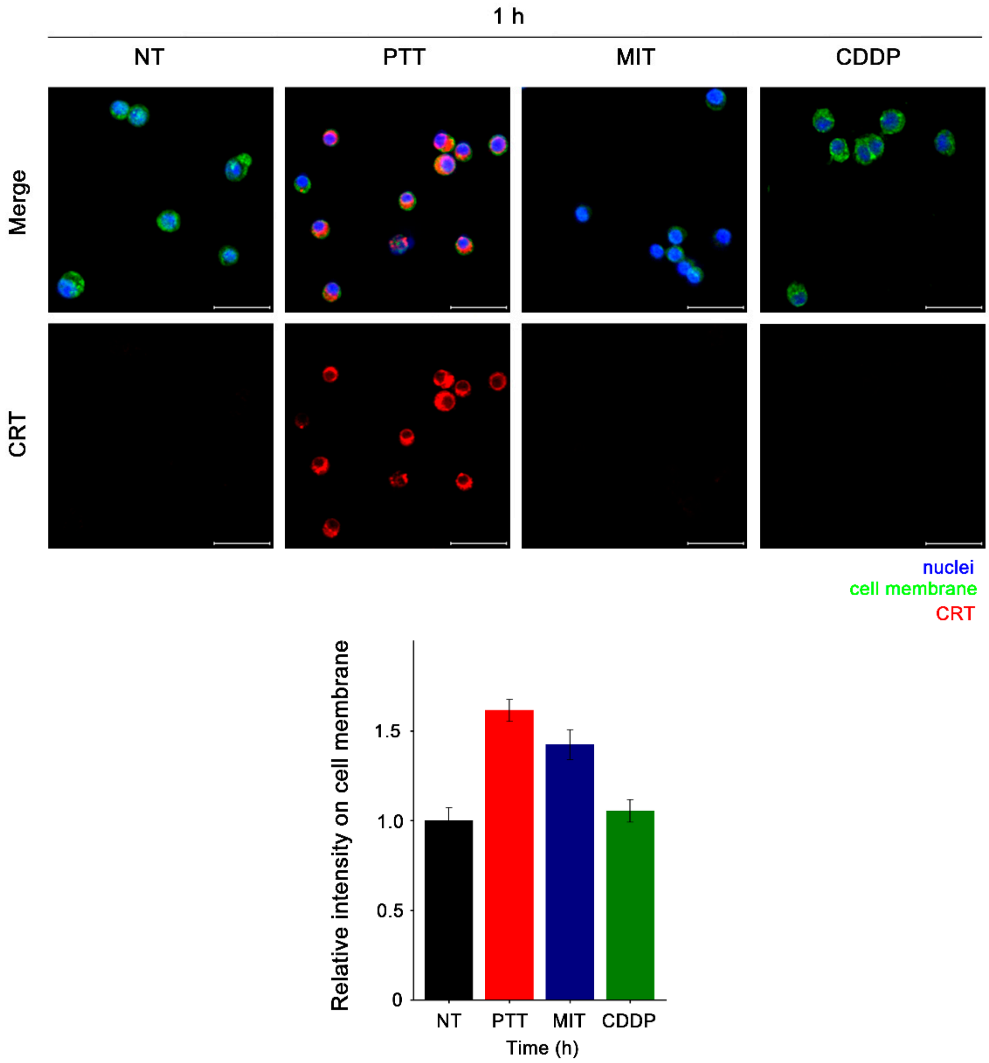

Figure S8. In vitro immunofluorescence of CRT

To quantify the calreticulin (CRT) expression on the cell membrane, the CRT on the cell membrane was visualized. MC38 cells were treated with PTT at 50–55°C for 10 min, or MIT (5 μM), or CDDP (100 μM), followed by incubation for 1h. Cells were fixed, permeabilized, and stained with anti-CRT (Red) and DAPI (Blue). Scale bar: 30 μm. Data are presented as mean ± S.E.; n = 153～321.

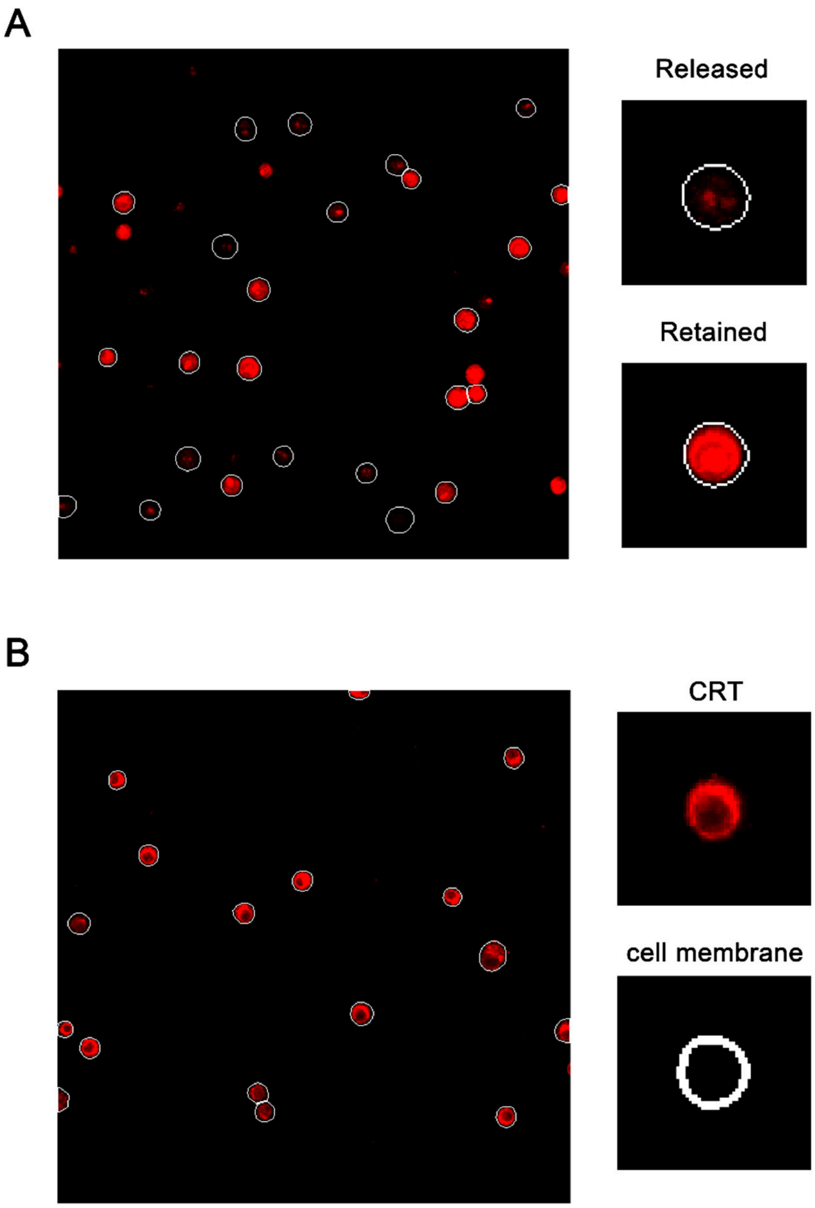

Figure S9. Method for quantification of ICD induction

(**A**) Cellular regions were assigned as the region of interest (ROI) using Cellpose 3.0. The percentage of the HMGB1-positive area in the ROI was then calculated. (**B**) The fluorescence intensity of CRT on the cell membrane was calculated.
